# Comparison of the epidemiology, risk factors, outcome and degree of organ failures of patients with candidemia acquired before or during ICU treatment

**DOI:** 10.1186/cc11307

**Published:** 2012-04-18

**Authors:** Pekka Ylipalosaari, Tero I Ala-Kokko, Juha Karhu, Markku Koskela, Jouko Laurila, Pasi Ohtonen, Hannu Syrjälä

**Affiliations:** 1 Department of Infection Control, Oulu University Hospital, Kajaanintie 50, Oulu, FIN-90029 OYS, Finland; 2 Department of Anesthesiology, Division of Intensive Care, Oulu University Hospital, Kajaanintie 50, Oulu, FIN-90029 OYS, Finland; 3 Departments of Anaesthesiology and Surgery, Oulu University Hospital, Kajaanintie 50, Oulu, FIN-90029 OYS, Finland; 4 Department of Clinical Microbiology, Oulu University Hospital, Kajaanintie 50, Oulu, FIN-90029 OYS, Finland

## Abstract

### Introduction

The aim of this study was to compare the epidemiology, risk factors, severity and outcome of two types of ICU-treated candidemias: namely, ICU-acquired candidemia (acquired after 48-hour ICU stay) (ICUAC group), and those needing ICU treatment for candidemia acquired before ICU admission or during the first 48-hour ICU stay (non-ICUAC group).

### Methods

A retrospective cohort study was conducted between 2000 and 2009 in a mixed tertiary ICU among patients with blood-culture-confirmed candidemia.

### Results

The study involved 82 patients (53 men). The ICUAC group consisted of 38 patients (46.3%) and the non-ICUA group included 44 patients (53.6). The ICUAC group had undergone previous surgery more often and had ICU stays that were 3.7 times longer than the non-ICUAC group, whose members more often had co-morbidities (95.6% versus 73.7%, *P *= 0.001). The ICUAC group had significantly more frequent organ failures with cardiovascular, renal, central nervous and coagulation systems than the non-ICUAC group. ICU, hospital and one-year mortality rates did not differ between the groups (23%, 36.8% and 65.8%, respectively, in the ICUAC group and 26%, 44.4% and 64.4%, respectively, in the non-ICUAC group). Among patients with APACHE II scores greater than 25, the ICUAC group had lower one-year mortality (65.0% versus 87.5%). Among patients with APACHE II scores of 25 or less, the ICUAC group had higher mortality (66.7% versus 50.0). *Candida albicans *was most common cause of candidemia in both groups (76.3% and 68.9%, respectively).

### Conclusions

More than half of the ICU-treated candidemias were acquired prior to admission to the ICU. Patients with ICU- and non-ICU-acquired candidemias had different risk factors and different needs for ICU resources. Hospital mortality was similar in both groups; however, the groups had different mortality rates when the severity of disease and underlying diseases were taken into account.

## Introduction

Candida species are among the most common microbes causing severe infections in patients in ICUs. Recent European publications of the causes of bloodstream infections (BSI) have listed Candida species as between the fifth and tenth most common causative pathogen [1]. Candidemia is associated with increased overall morbidity and attributable mortality, longer duration of ICU stay, as well as increased cost [2-5].

*C. albicans *has been the most common isolate (60% to 70%) among patients with ICU-acquired candidemia, followed by *C. glabrata *(15% to 20%). Some publications have shown an increase of candidemias with non-albicans *Candida *species, although contrary results have also been published [4,6-10].

Among ICU patients, candidemia is typically a late-onset nosocomial infection. As far as can be determined, few studies have investigated the characterization types of candidemias in the ICU. Specifically, this refers to candidemias acquired prior to ICU admission (non-ICUAC group) or within 48 hours of admission and those who have an ICU-acquired candidemia (ICUAC group), that is, more than 48 hours after ICU admission [1,11]. The present study examined whether these two candidemia groups have different risk factors, severity, outcomes or resource utilization or the spectrum of *Candida *species.

## Materials and methods

### Study location and design

The study was conducted at Oulu University Hospital, a 900-bed tertiary-level teaching hospital. The mixed medical-surgical ICU is an 11-bed unit with three two-bed rooms, one three-bed room and two single-bed rooms. This ICU has between 750 and 800 annual admissions.

Data from patients admitted into the medical-surgical ICU between 2000 and 2009 were evaluated retrospectively. The list of patients with positive blood cultures for Candida species was obtained from the register of the Clinical Microbiology Laboratory of Oulu University Hospital. Patients were divided into two groups: the non-ICUAC group, in which candidemia was diagnosed before ICU admission or during the first 48 hours after ICU admission; and the ICUAC group, in which candidemia was diagnosed more than 48 hours after ICU admission. We consulted the chief of the Ethical Committee and she noted that because the study design was a register-based survey with no contact or consequences to the patients approval from the Ethical Committee was unnecessary.

### Study parameters

The following information was collected for all study patients: age, gender, severity of underlying diseases, organ dysfunctions on admission as assessed by the Acute Physiology and Chronic Health Evaluation index (APACHE II) [12] and Sequential Organ Failure Assessment Score (SOFA) with six different types of organ failure [13], smoking habits, alcohol or drug abuse, presence of ischemic heart disease, chronic obstructive pulmonary disease (COPD), diabetes mellitus or hepatic failure, underlying hematological or other malignancy, recent use of immunosuppressive therapy, elective or emergency operations during the preceding 30 days, and previous antimicrobial therapy. The length of stay in the ICU and in the hospital was recorded, as was ICU and hospital mortality. Post-discharge mortality data were obtained from the Central Statistical Office of Finland on 28 June 2010.

### Microbiological analysis

Blood cultures were carried out by an automated continuous monitoring screening system of BacT/Alert (bio Mérieux, Marcy-l'Etoile, France). Between 2000 and 2005, 10 ml of venous blood was taken into standard aerobic and anaerobic blood culture bottles if neither antimicrobials nor antineoplastics were used during the preceding seven days; otherwise, the samples were cultured in in bottles containing neutralizing agents (Ecosorb) for antimicrobials. Corresponding, new FA/FN bottles have been used since 1999 for all patients suspected of having candidemia, and for all patients, since 2006. Until the end of October 2008, blood samples were drawn at the time clinical sepsis was documented and from between 30 minutes and two hours thereafter. From November 2008, both sets of blood samples were taken at the same time but from different blood vessels. The blood bottles were screened for up to five days and, in case there was a suspicion of slowly growing bacterial and yeast pathogens, up to 14 days. Positive blood cultures were gram-stained and subcultured. Commercial kits identified yeast growth as *C. albicans *or yeast, *not albicans*, within a few hours and further as species level within two days by a API ID 32 C (bioMérieux). Positive blood cultures were gram-stained and subcultured. Commercial kits identified yeast growth as *C. albicans *or non- *albicans *within a few hours, and further as species level within two days. E-test was used to test antifungal susceptibility to fluconazole, voriconazole, caspofungin and amphotericin B. Bacterial and yeast findings were also reported immediately by phone to the clinician.

### Data registration and statistical analysis

The data were entered into a SPSS database (SPSS Data Entry, version 2.0, SPSS Inc., Chicago, IL). Summary statistics for continuous or ordinal variables are expressed as the median with the 25^th ^to 75^th ^percentile. Kaplan-Meier survival curves and log-rank test results are presented for simple comparisons between non-ICU and ICU-acquired candidemia groups. The APACHE II score (< 25 or > 25) was used as an adjusting factor in Cox's proportional hazards model to further evaluate the group effect in one-year mortality. Two-tailed *P*-values are reported, and the analyses were performed by the SPSS (version 18.0, SPSS Inc., Chicago, IL) software.

## Results

### Characteristics of study groups

The total study population consisted of 82 patients with candidemia: 38 in the ICUAC group and 44 in the non-ICUAC group. The incidence of ICUAC per 1,000 patient days varied from 0.19 (in 2005) up to 0.9 (in 2000 and 2009). The proportion of patients with non-ICUAC slightly increased during the study years, from 50% (19/38) during the first five-year period to 58% (26/44) during the last five years.

Table 1 presents the main demographic data of the groups. Patients in the non-ICUAC group more often had underlying co-morbidities (96% versus 46%, *P *= 0.01), especially malignancies (29% versus 11%, *P *= 0.04). As Table 1 also shows, patients in the non-ICUAC group had experienced recent surgery significantly less often (60% versus 84%, *P *= 0.016) and they also had central venous catheters (64%) less often than patients in the ICUAC group (100%, *P *< 0.001). In both study groups, the median time between previous surgery and candidemia was one week; gastrointestinal surgery (39% of patients) was most common, followed by plastic surgery (8% of patients).

**Table 1:** Main demographic data in the ICUAC and non-ICUAC groups.

	ICUACG *n *= 38	Non-ICUACG *n *= 44	*P *value
Male (%)	27 (71)	27 (61)	0.35
Age years	63 (45 to 69)	64 (56 to 75)	0.194
Co-morbidities (%)	28 (73.7)	43 (95.6)	0.01
Distribution (%)			
- Diabetes	5 (13.2)	7 (15.6)	> 0.9
- Malignancy	4(11)	13 (29)	0.04
- COPD	4 (1.5)	6 (13.3)	> 0.9
- Hepatic cirrhosis	1 (2.4)	4 (8.9)	0.39
- IHD	9 (23.7)	16 (35.6)	0.337
- Alcohol abuse	9 (32.1)	7 (20.6)	0.386
Previous surgery (%)	32 (84.2)	27 (60)	0.016
- Intra-abdominal surgery	16 (50)	16 (39)	0.47
Central venous catheter (%)	38 (100)	29 (64)	< 0.001
Duration of central venous catheter before positive BC (days)	7 (6 to 11)	7 (6 to 7)	0.84
Previous antibacterial treatment (%)	37 (97.4)	43 (95.5)	> 0.9
Previous fluconazole prophylaxis (%)	15 (39.5)	6 (13.3)	0.010

Values are presented as median (with 25^th ^to 75^th ^percentiles) or as the number (percentage) of patients. BC, blood culture; COPD, chronic obstructive pulmonary disease; IHD, ischemic heart disease; TIA, transient ischemic attack.

Patients in the ICUAC group had received pre-emptive fluconazole treatment significantly more often (13.3% versus 40%, *P *= 0.007). Most patients had also been on antibacterial treatment before candidemia. The most common antibacterials used in the ICUAC group and the non-ICUAC group were as follows: piperacillin/tazobactam in 22 (58%) and 24 patients (52%), second generation cephalosporins in 15 (39%) and 19 patients (41%), fluoroquinolones in 15 (39%) and 6 (13%) patients, and carbapenems in 8 (21%) and 6 patients (13%), respectively.

As Table 2 shows, the median APACHE II or SOFA scores did not differ statistically between the groups. However, when six different organ failures were compared between the two candidemia groups (Table 3), the patients in the ICUAC group had significantly higher maximum SOFA scores in coagulation (*P *= 0.012), cardiovascular (*P *= 0.010), central nervous system (*P *= 0.000) and renal systems (*P *= 0.005). The median duration of hospital stay before ICU admission was 1.0 day (with 25 and 75 percentiles 0.0 to 2.0) in the ICUAC group and 7.5 days (1.0 to 16.0) in the non-ICUAC group. The median duration of hospital stay before positive blood cultures was 12 days (7.0 to 19.0) in the ICUAC group and 11 days (3.0 to 17.0) in the non-ICUAC group.

**Table 2:** Severity scores on the day of positive blood cultures in the ICUAC and non-ICUAC groups.

	ICUACG *n *= 38	Non-ICUACG *n *= 45	*P*-value
APACHE II on admission	27 (18 to 29)	23.5 (18.5 to 29)	0.79
APACHE II on the day when BC positive	20.5 (16.5 to 26)	22.5 (19 to 27)	0.34
SOFA on admission	9 (7 to 11)	8.5 (5 to 11)	0.281
SOFA on the day when BC positive	10 (7 to 13.5)	7 (5 to 11)	0.172

Values are presented as a median (with 25^th ^to 75^th ^percentile) or as the number (%) of patients. APACHE II, Acute Physiology and Chronic Health Evaluation score; BC, blood culture; ICUACG, ICU-acquired candidemia group; non-ICUACG, non-ICU-acquired candidemia group; LOS, length of stay; SOFA, Sequential Organ Failure Assessment scale.

**Table 3:** Comparison of the highest SOFA-scores in the ICUACG and the non-ICUACG.

Parameter	ICUACG *n *= 38	Non-ICUACG *n *= 45	*P*-value
Respiratory	3 (2 to 4)	3 (2 to 3)	0.064
Coagulation	2 (1 to 3)	1 (0 to 2)	0.012
Liver	2 (1 to 3)	1 (0 to 3)	0.097
Cardiovascular	4 (3 to 4)	3 (3 to 4)	0.010
CNS	3 (2 to 4)	1 (0 to 3)	0.000
Renal	4 (0 to 4)	1 (0 to 3)	0.005

Values are presented as medians (with 25^th ^to 75^th ^percentiles). CNS, central nervous system; ICUACG, ICU-acquired candidemia group; non-ICUACG, non-ICU-acquired candidemia group; SOFA, Sequential Organ Failure Assessment scale.

### Laboratory data and clinical characteristics

The ICUAC group had a higher body temperature on the day of positive blood culture than the non-ICUAC group (38.8°C versus 38.3°C, *P *= 0.03). On the other hand, C-reactive protein (CRP) and leukocyte counts on the day of positive blood culture did not differ between the groups (CRP was 126 mg/L in the ICUAC group and 165 mg/L in the non-ICUAC group (*P *= 0.7) and leukocyte count was 11.3 x10^9^/L versus 9.4 x10^9^/L, respectively). Abnormal renal function (creatinine > 90 mmol/L in women and > 100 mmol/L in men) was noticed in 16 patients (42%) in the ICUAC group and in 23 patients (49%) in the non-ICUAC group. In the ICUAC group, CRP reached its peak on day three after positive blood culture: 160 mg/L (with 25 percentile of 100 mg/L and 75 percentile of 232 mg/L) while CRP in the non-ICUAC-group peaked on the day of blood culture at 165 mg/L (with 25 percentile of 61 mg/L and 75 percentile of 195 mg/L).

### Distribution of candida species

*C. albicans *was the most common isolate in both groups across the entire study period (Table 4). The proportion of non-albicans species was 26.7% in the ICUAC group and 31.1% in the non-ICUAC group. The most common non-albicans species was *C. glabrata*. There was a relatively small and non-significant decrease of non-albicans species in the ICUAC candidemia group between the first five years and the last five years (26.3% versus 21.1%, respectively) and, on the contrary, a small non-significant increase in the non-ICUAC group (26.3% versus 34.6%, respectively).

**Table 4:** Distribution of Candida species in the ICUACG and non-ICUACG groups.

	ICUACG *n *= 38	Non-ICUACG *n *= 45	*P*-value
*C. albicans *	29 (76.3)	31 (68.9)	0.47
*C. non- albicans *	9 (26.7)	14 (31.1)	
*- C. glabrata*	5	10	
*- C. parapsilosis*	3	2	
- others	1	2	
*C. albicans*			
- 2000 to 2004, *n *= 37	14 (73.7)	14 (77.8)	> 0.9
- 2005 to 2009, *n *= 45	15 (78.9)	17 (65.4)	
*C. non- albicans*			
- 2000 to 2004	5 (26.3)	5 (26.3)	0.51
- 2005 to 2009	4 (21.1)	9 (34.6)	

Other species were: *C. guillerimondii, C. krusei *and *C. tropicalis*. ICUACG, ICU-acquired candidemia group; non-ICUACG, non-ICU-acquired candidemia group.

### Antifungal treatment

The median duration of the antifungal treatment was 22 days in patients in the ICUAC group and 24 days in the non-ICUAC group. The most common antifungal used was fluconazole (38 patients (73%) in the ICUAC group and 35 (77%) in the non-ICUAC group), followed by liposomal amphotericin (13 patients (34%) in the ICUAC group and 16 patients (35%) in the non-ICUAC group) and echinocandins (12 patients (31%) and 18 patients (40%), respectively). The median time between positive blood cultures and the beginning of antifungal treatment was less than 24 hours (with 25^th ^percentiles 0 to 2-days in both groups) and there were no differences between the groups. Patients with fluconazole prophylaxis were not included in this analysis. Eighteen patients out of 82 (23% of the whole study population) developed breakthrough candidemia during fluconazole treatment. Of these 18 patients, 16 (89%) had previously been operated on and two (11%) had malignancies. The median duration of the fluconazole prophylaxis was 7.0 days.

Fluconazole-resistant *C. albicans *or *glabrata *was found in ten patients, six of whom were in the non-ICUAC group and four in the ICUAC group. Four out of ten patients had previously received fluconazole. Hospital mortality was significantly higher among patients with candidemia due to fluconazole-resistant candida than patients with candidemia due to fluconazole-sensitive candida (90% versus 37%, *P *< 0.002).

### Outcome

As Table 5 shows, the length of hospital stay did not differ between the groups. The median duration of hospital stay before ICU admission was 1.0 day (with 25th to 75th percentiles 0.0 to 2.0) in the ICUAC group and 7.5 days (1.0 to 16.0) in the non-ICUAC group (*P *< 0.0001). The median duration of hospital stay before positive blood cultures was 12 days (7.0 to 19.0) in the ICUAC group and 11 days (3.0 to 17.0) in the non-ICUAC group. Among the patients in the ICUAC group, the time between ICU admission and the onset of candidemia was 8.0 days (1.0 to 12.0).

**Table 5:** The length of ICU and hospital stay and mortality data in the ICUACG and the non-ICUACG.

	ICUACG *n *= 38	Non-ICUACG *n *= 45	*P*-value
Length of hospital stay (days) in surviving patients	38.0 (22 to 59)	40.0 (22 to 57)	0.762
Length of ICU stay (days) in surviving patients	16.0 (11 to 30)	4.0 (2 to 9)	< 0.001
			
ICU mortality	8 (23)	12 (26)	
28-day mortality	8 (21.1)	14 (31.1)	0.33
Hospital mortality	14 (36.8)	20 (44.4)	0.51
One-year mortality	25 (65.8)	29 (64.4)	> 0.9

ICUACG, ICU-acquired candidemia group; non-ICUACG, non-ICU-acquired candidemia group.

**Figure 1:**
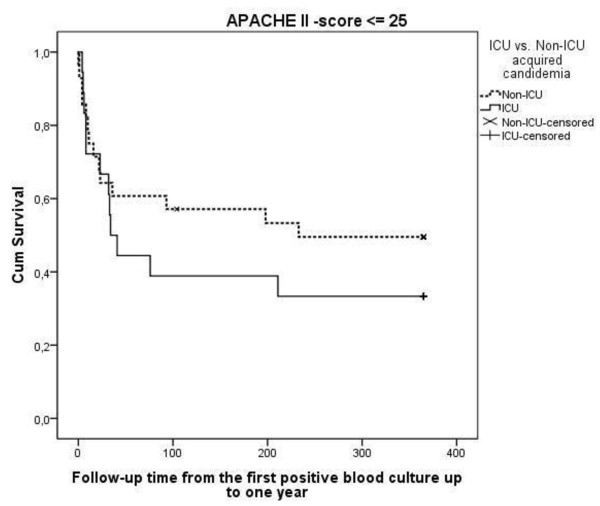
**Kaplan-Meier one-year survival curves in ICUACG (ICU acquired candidemia group) and the non-ICUACG (non-ICU acquired candidemia group) for patients with APACHE II (Acute Physiology and Chronic Health Evaluation index) scores on admission of ≤ 25**. The APACHE II score adjusted *P*-value according to log-rank test is 0.60.

**Figure 2:**
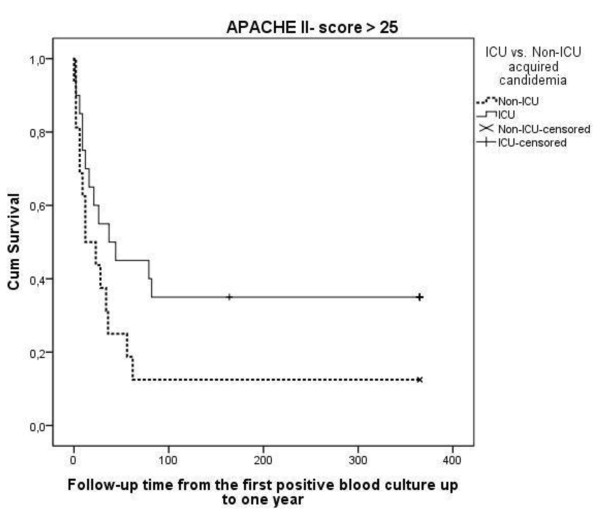
**Kaplan-Meier one-year survival curves in ICUACG (ICU acquired candidemia group) and the non-ICUACG (non-ICU acquired candidemia group) for patients with APACHE II scores on admission > 25**. The APACHE II score adjusted *P*-value according to log-rank test is 0.60.

The length of stay in the ICU was significantly longer among the surviving patients with ICU-acquired infection (Table 4). The ICU mortality did not differ greatly between the groups (23% versus 26%), and the same applies for hospital mortality (36.8% versus 44.4%) and one-year mortality (65.8% versus 64.4%). The majority of the deaths (55.4%) occurred during the first 60 days after positive blood cultures in both groups (Figure 1). In the ICUAC group, one-year mortality among patients who had APACHE II scores of 25 or less was higher (66.7% versus 50.0%, Figure 1), while the mortality was lower among patients with APACHE II scores greater than 25 (65.0% versus 87.5%, Figure 2). This result was supported by the multivariate Cox model with the interaction term (group x APACHE II score HR 2.7; 95% confidence interval 0.92 to 8.1).

## Discussion

The results of this study show that more than half of the candidemia patients treated in the ICU acquired the infection before ICU admission and more often had co-morbidities, especially malignancies. On the other hand, the real ICU-acquired candidemia patients had undergone surgery more often and they all had central lines. Moreover, ICUAC patients had statistically higher maximum SOFA scores in four of six organ systems.

This study has several strengths. Earlier literature of ICU-treated candidemia did not compare the epidemiology and outcome of non-ICU onset and ICU-acquired candidemias. The case definition required blood culture confirmation for candidemia. All blood cultures had been processed in the same microbiological laboratory, which is accredited by the Finnish Lab Quality-system. Moreover, the study followed the long-term outcome of candidemia patients for up to one year. The study also has some limitations. Due to its retrospective approach, some essential information was probably overlooked. Moreover, the study was only performed in a single mixed ICU, so the results cannot be directly extrapolated to other types of intensive care units. Furthermore, the relatively small study groups did not seem to justify the use of multivariate analysis for the risk factors of mortality.

The recent literature includes some reports of increasing figures of the overall incidence of candidemia, as well as ICU-acquired candidemia since 2000 [3,8]. In the present series, the incidence of ICU-acquired candidemia was quite stable during the relatively long study period. Data from the National Nosocomial Infection System (NNIS) survey showed a decline in the frequency of candidemia among ICU patients in the United States, while the burden of invasive candidiasis is shifting from the ICU to the general hospital wards [14]. Another report from Italy also concluded that candidiasis can no longer be considered to be just an ICU-related infection and that efforts to design preventive and diagnostic strategies must be expanded to include other at-risk populations and hospital environments [15]. Recent literature has also reported that nearly one-quarter of candidemias were healthcare-associated [16]. These findings are in keeping with ours, where less than half of the patients needing ICU treatment due to candidemia belonged to the ICUAC group. In our series, ten out of 45 patients (22%) in the non-ICUAC group had acquired healthcare-associated candidemia before admission (data not shown). Besides, the number of patients in the non-ICUAC group was almost 1.5 times higher during the last five-year period than in the first five-year period of the study. One explanation for this could be more active use of blood cultures for the diagnosis of infections in the wards of the studied hospital, where the number of obtained blood cultures increased 2.3-fold from 2000 to 2009 (according to the register of the Microbiology Laboratory).

As far as can be ascertained, few publications have compared ICU-acquired candidemia patients with non-ICU-acquired candidemia cases needing ICU treatment. For example, a large multicenter study with 300 ICU-treated patients in French ICUs did not reveal this specific topic [17]. In the present series, the ICU stays of the ICUAC group were 3.7 times longer than patients in the non-ICUAC group. Moreover, the ICUAC group had statistically higher maximum SOFA scores concerning coagulation, cardiovascular, central nervous and renal systems. When the two time periods were compared, similar results concerning the circulation and renal systems were observed during the last period.

The risk profiles for candidemia were also different in those groups that had an impact, for example, on prevention of those infections. Patients with ICU onset candidemia had a history of previous surgery significantly more often, especially intra-abdominal operations. In addition, they all had central venous catheters. On the other hand, patients in the non-ICUAC group more often had underlying diseases, such as malignancies. The risk of candidemia in those patients is more probably due to the immunosuppressive condition, which is often hard to influence by infection control methods [18].

Despite high severity scores on admission, the hospital mortality of candidemia patients treated in the ICU was only 43.4% in the present series, with the similar rates in both groups (36.8% versus 44.4%) being almost identical to the mortality rate of 42.6% in the EPIC II study [19]. Hospital mortality in other studies has varied from 26% to 62% among patients with *C. albicans *fungemia, and from 34% to 90% among patients with *non-albicans *candidemia [6,11,20]. Delayed administration of antifungals had been reported to be a risk factor for worse outcome [21-23]. In the present series, the median time between taking positive blood cultures and starting antifungal treatment was less than 24 hours in both survey groups. Another explanation of favorable outcome could be the relatively low proportion of non-albicans species. It is important to note that although almost 40% of patients in the ICUAC group had prophylactic or pre-emptive treatment with fluconazole, they developed a breakthrough invasive candida infection. Breakthrough candemia during fluconazole treatment has been reported, for example, in association with hematological malignancies [24]. Similar breakthrough invasive candidal infections have even been reported during echinocandin treatment [25-27].

The existing literature has sparse information about the long-term outcome of candidemia patients. In a nationwide survey of candidemia in Israel, one-year mortality was 31.9% [28] and 90% of patients died within 90 days after the identification of candidemia. In the present ICU series, one-year mortality was twice as high (65.9%), while 60.2% of the deaths occurred within 90 days. Non-ICUAC patients with APACHE scores greater than 25 on admission had higher long-term mortality which reflects the importance of underlying co-morbidities in survival.

The trend of changes in the distribution of *C. albicans *and non-*albicans *species has been controversial in the recent literature. In a recent study from Brazil, the proportion of non-*albicans *species was exceptionally high, at 82% [29]. In harmony with most other recent publications, *C. albicans *was the most common species in the present study population. In fact, there was even a small but insignificant decrease of non-*albicans *species in the ICUAC candidemia group and a small increase of these species in the non-ICUAC group. It has been noted that even a three-year period with routine fluconazole prophylaxis for selected Surgical ICU patients did not cause an increase of *C. glabrata *colonization or invasive candidasis due to *C. glabrata *[30].

In conclusion, the present study shows that the majority of ICU treated candidemias are actually acquired before ICU admission. The ICUAC group had more specific organ failures than the non-ICUAC group. Moreover, the groups had different outcome profiles when the patients were categorized according to APACHE II scores. Non-ICUAC patients more often had underlying co-morbidities, whereas ICUAC candidemia patients had more often undergone previous surgery and all had central venous lines. Taken together, future studies should account for the fact that ICUs are treating two different groups of candidemia patients.

## Key messages

• More than half of the ICU-treated candidemias are actually acquired before ICU admission. *Candida albicans *is still the most common cause of candidemia.

• Patients with non-ICU-acquired candidemia have different risk factors than patients with ICU onset candidemia.

• Patients in the ICUAC group had significantly higher maximum SOFA scores in coagulation, cardiovascular, central nervous system and renal systems than patients in the non-ICUAC group.

## Abbreviations

APACHE: Acute Physiology and Chronic Health Evaluation index; ARDS: acute respiratory distress syndrome; BC: blood culture; COPD: chronic obstructive pulmonary disease; ICUAC: intensive care unit acquired; IHD: ischemic heart disease; LOS: length of stay; SOFA: Sequential Organ Failure Assessment scale; TIA: transient ischemic attack

## Competing interests

The authors declare that they have no competing interests.

## Authors' contributions

PY participated in the design of the study and collection and analysis of data and drafted the manuscript, JK participated in the collection of data. TA-K, JL, and HS participated in the design of the study and the analysis of data and drafted the manuscript, MK participated in the design of the study and analysis of the microbiological samples and drafted the manuscript, PO participated in the design of the study and performed the statistical analysis. All authors read and approved the final manuscript.
